# The effect of weighted blankets on sleep and related disorders: a brief review

**DOI:** 10.3389/fpsyt.2024.1333015

**Published:** 2024-04-15

**Authors:** Jie Yu, Zhenqing Yang, Sudan Sun, Kaili Sun, Weiran Chen, Liming Zhang, Jiahui Xu, Qinglin Xu, Zuyun Liu, Juan Ke, Lisan Zhang, Yubo Zhu

**Affiliations:** ^1^ Center for Clinical Big Data and Analytics of the Second Affiliated Hospital, and Department of Big Data in Health Science School of Public Health, The Key Laboratory of Intelligent Preventive Medicine of Zhejiang Province, Zhejiang University School of Medicine, Hangzhou, China; ^2^ Department of Neurology/Center for Sleep Medicine, Sir Run Run Shaw Hospital, School of Medicine, Zhejiang University, Hangzhou, China; ^3^ Department of Internal Medicine of Traditional Chinese Medicine, Shaoxing Hospital of Traditional Chinese Medicine, Shaoxing, China; ^4^ Department of Neurology, Affiliated Hospital of Shaoxing University, Shaoxing, China

**Keywords:** weighted blanket, sleep disorder, insomnia, deep pressure, occupational therapy, psychiatric disorder

## Abstract

**Background:**

Sleep disorders such as insomnia can lead to a range of health problems. The high risk of side effects and drug abuse of traditional pharmacotherapy calls for a safer non-pharmacotherapy.

**Aims:**

To examine the use and efficacy of weighted blankets in improving sleep and related disorders in different populations and explore the possible mechanisms.

**Methods:**

A literature search was conducted using PubMed, Embase, Web of Science, MEDLINE, Cochrane Library and CNKI databases. Eligible studies included an intervention with weighted blankets and outcomes covering sleep and/or related disorders (behavioral disturbance, negative emotions and daytime symptoms). Studies using other deep pressure, compression, or exercise-related interventions were excluded.

**Conclusions:**

Most of the included studies showed that weighted blankets could effectively improve sleep quality and alleviate negative emotions and daytime symptoms in patients with sleep disorders, attention deficit hyperactivity disorder, autism spectrum disorder, and other related disorders, with a possible mechanism of deep pressure touch.

**Recommendations:**

Weighted blankets might be a promising tool for sleep interventions among individuals with sleep disorders in clinical settings. More high-quality and large-scale randomized controlled trials are needed to further validate the safety and efficacy of weighted blankets and explore precise mechanisms.

## 1 Introduction

Quality sleep is essential for psychological, cardiovascular, metabolic, and other aspects of health (1). However, during the COVID-19 pandemic, with about 40% of people reporting sleep problems, sleep-related problems have become more severe and were particularly prominent in some populations, including children and adolescents (2, 3). Sleep disorders, such as insomnia, are common risk factors for many psychiatric disorders and may also be a symptom of mental health disorders (4, 5). In any case, effective interventions for insomnia should be provided to prevent the development of sleep disorders and alleviate other mental health problems (6, 7). Common treatments for insomnia are mainly pharmacotherapy and cognitive behavioral therapy (non-pharmacotherapy), with the latter being preferred for its safety and durability (8, 9). However, about 40% of patients with persistent insomnia do not respond to cognitive behavioral therapy combined medication treatment (10). In addition, pharmacotherapy for insomnia often leads to a high risk of side effects and drug abuse. In adverse drug reaction studies, the highest reporting odds ratios of somnolence are 7.1 and 13.3 for patients prescribed antidepressants (11) and antipsychotics (12), respectively. For patients prescribed higher dose Z-drugs, the hazard ratios of fractures, hip fractures, ischaemic stroke, and falls ranged from 1.33 to 1.96 (13). Thus, a safer non-pharmacotherapy needs to be explored.

Weighted blanket, an emerging non-pharmacotherapy, has gradually entered people’s vision. A weighted blanket is usually made of special weight-adding materials such as beads and chains, allowing it to cover the body evenly and create a pleasant hugging sensation. As a non-invasive intervention assistive device, a weighted blanket was initially suggested as a prescription or treatment option by occupational therapists for patients with sleep problems (14). To date, weighted blankets have been increasingly used in sleep interventions for different populations, such as patients with insomnia, attention deficit hyperactivity disorder (ADHD), and autism spectrum disorder (ASD), and have shown positive effects on relieving insomnia, anxiety, and fatigue (15, 16). Only one previous review has evaluated the effect of weighted blankets on decreasing anxiety and insomnia using 8 studies conducted before March 1, 2018 (17). However, the use of weighted blankets for wider targets has surged in recent years, calling for an updated and comprehensive review of the effect of weighted blankets in different populations and the in-depth mechanisms.

Therefore, this narrative review summarized the use and efficacy of weighted blankets in sleep and other related disorders (e.g., behavioral disturbance, negative emotions and daytime symptoms) in different populations and discussed the relevant mechanisms to provide a reference for a comprehensive overview of the research status of weighted blankets and make recommendations for an alternative therapy to medications of sleep and sleep-related disorders in clinical settings.

## 2 Materials and methods

We searched in PubMed, Embase, Web of Science, MEDLINE, Cochrane Library and CNKI using optimized search strategies up to March 10, 2023. We determine the search terms using appropriate controlled vocabulary terms. The final search terms consisted of “weighted blankets”, “gravity blankets”, “ball blankets”, “sleep”, and “sleep quality”. Studies were limited to those published in English.

A study was included if it met the following criteria: (1) it included an intervention with weighted blankets; (2) it reported parameters that assessed the sleep quality (e.g., sleep latency, sleep duration, sleep efficiency, and number of wakings) and/or related disorders (e.g., behavioral disturbance, negative emotions and daytime symptoms). A study was excluded if it used other deep pressure, compression, or exercise-related interventions. Furthermore, letters, study protocols, guidelines, dissertations, and thesis were excluded.

We read the titles and abstracts to identify all relevant studies using the inclusion and exclusion criteria above. Then, two reviewers worked independently to assess each included study and resolved any discrepancies through discussion (see flow chart in **Supplementary Figure 1**).

## 3 Results

### 3.1 Application of weighted blankets in improving sleep

Sleep disturbance, a symptom or harbinger of psychiatric problems, can be comorbid with psychiatric disorders and increases the risk of psychiatric relapse (18). To improve sleep quality and reduce the negative impact of sleep disturbance on psychiatric disorders, occupational therapists have attempted to use a weighted blanket as a safer physical therapy (19). A randomized controlled trial in Sweden has provided primary evidence for weighted blankets’ efficacy in relieving insomnia and daytime fatigue in patients with psychiatric disorders (16). Besides, another randomized controlled study showed that after using weight blankets, insomniacs had improved sleep quality, shorter nighttime awakenings, reduced self-reported stress, and enhanced relaxation (20). For older people living in nursing homes, weighted blankets also have positive effect on improving sleep especially concerning waking up during the night (21). Furthermore, weighted blankets have also been used to treat sleep disorders in children with ADHD, ASD, and CHARGE syndrome (22–24). Interestingly, a review on non-traditional clinical treatments for improving sleep in children and young people suggested that although the weighted blankets intervention did not result in significant differences in sleep indicators, there was positive feedback on self-reports from subjects and caregivers (23). This finding revealed a difference in the efficacy between subjective and objective sleep measurements. Non-significant effects on subjective sleepiness and total sleep duration were observed in an in-laboratory crossover study. However, the significant increase of salivary melatonin in this study provided new evidence for the sleep-promoting mechanisms of weighted blankets (25).

### 3.2 Application of weighted blankets in ADHD and ASD

Several studies have used weighted blankets as an intervention for patients with neurodevelopmental disorders, including ADHD and ASD. Most of them indicated a beneficial impact of weighted blankets on the improvements in sleep quality (e.g., sleep onset latency) and ADHD/ASD-related symptoms (e.g., concentration and daily function) in patients with AHDH or ASD (**Table 1**).

**Table 1:** Application of weighted blankets in attention deficit hyperactivity disorder (ADHD) and autism spectrum disorder (ASD).

Country	Sample characteristics	Study design (intervention duration)	Primary outcomes	Effect
Danish (26)	21 children aged 8–13 years with ADHD vs 21 healthy control subjects	Case-control study (14 days)	Reduced sleep onset latency*; Activity level (ns); Attention (ns); Behavioural disturbance symptoms (ns)	Positive
England (27)	67 children aged 5–16 years with ASD and severe sleep problems	Randomized, placebo-controlled crossover design (4 weeks)	Better subjective feeling*; Total sleep time (ns); Sleep-onset latency (ns); Sleep efficiency (ns)	Uncertain
USA (28)	Two children with ASD and sensory over responsivity	Single case, multiple baseline design (14 days)	Improvements in time to fall asleep, number of wakings, hours of sleep, and morning mood	Positive
USA (29)	Two children with ASD and sensory over responsivity	Intervention study (14 days)	Improvements in hours of sleep, morning mood, and waking times in the intervention phase; Reduced hours of sleep, a more agitated mood, and increased waking times in the withdrawal phase	Positive
Sweden (22)	85 individuals with ADHD and/or ASD	Retrospective follow-up study	Improved abilities related to falling asleep, sleeping the whole night, relaxing during the day; Improved morning/evening daily routine	Positive
Danish (30)	36 children aged 8–13 years with ADHD	Intervention study (8 weeks)	Improved sleep onset latency*; Reduced score on core symptoms of ADHD*; Increased daily level of functioning and the quality of life*	Positive
Switzerland (31)	24 parents of children with ADHD and sleep problems	Qualitative study (16 weeks)	Improvements in achieving satisfactory sleep; achieving overall well-being; mastering everyday life	Positive
Sweden (32)	1785 adult individuals with a psychiatric diagnosis	Population-based register study (12 months)	The proportion of patients without a prescription of sleep medication increased by 3.3%*; Melatonin prescription increased by 3.6%*; ADHD was associated with decreased use of sleep medication*	Positive
USA (15)	Two 4-year-old children with ASD	Single-subject design study (14 days)	Enhanced morning mood; Decreased time to fall asleep	Positive
Sweden (33)	26 children aged 6-15 years with ADHD and sleeping difficulties	Qualitative study (16 weeks)	Improvements in emotional regulation, sleep routines, sleep quality, and everyday participation	Positive

*P < 0.05.

ADHD, Attention Deficit Hyperactivity Disorder; ASD, Autism Spectrum Disorder; ns, non-significant.

Specifically, a case-control study found that after using a weighted ball blanket for 14 days, children aged 8-13 years with ADHD had improvements in sleep onset latency, awakening times, concentration, and physical activities (26). Another Danish study also provided evidence for the positive effects on sleep onset latency, daily functioning levels, and quality of life in children with ADHD in the same age range after using weighted ball blankets for 8 weeks (30). Similarly, A qualitative study based on parents’ experiences of the impact of weighted blankets on children with ADHD and sleep disorders indicated that after 16 weeks of weighted blankets intervention, children could better master everyday life, and achieve satisfactory sleep and overall well-being (31). Another qualitative study based on children’s experiences revealed that the use of weighted blankets improved the emotional regulation, everyday participation and sleep quality of children with ADHD and sleeping difficulties (33). Moreover, in Idaho, USA, a single-subject design study (28) and two continuation studies (15, 29) in children with ASD and sleep disorders indicated that weighted blankets were beneficial for the improvement of overall sleep quality, especially the sleep onset latency, awakening times, sleep duration, and morning mood, although the effects were not sufficient to recommend for clinical use. However, a randomized, placebo-controlled crossover design trial observed that the use of weighted blankets did not help children aged 5-16 years with ASD and severe sleep problems obtain longer sleep duration, faster sleep onset, or fewer awakenings (27).

In addition to children, a randomized controlled study in Stockholm, Sweden, found that in adult patients with ADHD, a weighted chain blanket was also a safe and effective intervention for insomnia, which could improve adult patients’ daytime symptoms and activity levels (16). Interestingly, a population-based registry study indirectly demonstrated the positive effect of weighted blankets intervention on reducing the use of common sleep medication among ADHD patients (32). Moreover, another registry study found that patients with ADHD retained the weighted blankets longer than others, indicating a possible benefit from using weighted blankets (34). Likewise, a retrospective follow-up study in 2021 found that after interventions of a weighted chain or ball blanket, both adults and children with ADHD or ASD showed improvements in sleep, daytime relaxation, and morning/evening routine (children significantly benefited more in falling asleep than adults.) (22).

### 3.3 Application of weighted blankets in improving negative emotions

There are 17 studies (including one systematic review) related to weighted blankets intervention and psychological behaviors (e.g., anxiety, depression, and stress). Two single-subject design studies found that patients with nighttime weighted blanket intervention had a better morning mood, which could be agitated and unstable when the intervention was discontinued (15, 29). A retrospective follow-up study showed that weighted blankets benefited daytime relaxation in patients with ADHD/ASD (22). Furthermore, a randomized controlled study showed a positive effect of weighted chain blankets on depression and anxiety symptoms (16). However, two similar conference abstracts argued that weighted blankets could not reduce stress in patients with pain (35, 36), while another conference abstract indicated that weighted blanket could reduce self-reported stress and increase relaxation in patients with sleep disorders (20).

A few studies have focused on the effect on alleviating anxiety. A systematic review suggested that weighted blankets might be an appropriate therapeutic tool for reducing anxiety (17). Additionally, a controlled clinical trial in the United States based on a repeated crossover design found that compared to standard care, the use of weighted blankets for 30 minutes resulted in significantly lower anxiety levels (based on the visual analog scale for anxiety) in cancer patients undergoing outpatient chemotherapy infusion (37). Significant alleviation in anxiety (measured by pulse and a shortened form of the Spielberger State-Trait Anxiety Inventory [STAI: Y-6]) was also found among adult patients in mental health institutions after using weighted blankets (38). Moreover, a pilot study at a liberal arts university in the Midwest suggested that a weighted blanket may improve sleep quality and reduce anxiety among college students (39). Similar results were also found in patients with ADHD/ASD. Two qualitative studies on nursing home staff (40) and children with ADHD and sleep problems (31) also provided positive evidence that weighted blankets helped relax and reduced anxiety.

### 3.4 Application of weighted blankets in special populations

#### 3.4.1 In patients with chronic pain

A total of three studie*s* were conducted on patients with chronic pain. A randomized controlled trial showed that heavier-weighted blankets produced a greater alleviation in widespread chronic pain than the lighter-weighted blankets, and the effects were stronger in individuals with high trait anxiety (41). However, two of the Danish conference abstracts (36, 42) indicated that no difference of HbA1C, a reflection of stress, was found between patients with chronic pain in the weighted ball blanket group and the control blanket group.

#### 3.4.2 In patients with dementia

Four studies have explored the effect of weighted blankets on patients with dementia. One intervention trial in female patients with late-stage of dementia found a significant reduction in the duration of sustained vocalizations after 10 min of weighted blanket intervention, suggesting that it was a promising non-pharmacological treatment for patients with dementia (43). Likewise, a prospective, within subjects, pre-post design study with a 4-week intervention indicated that weighted blankets were of high feasibility and acceptability for families living with dementia (44). Another case report in Japan demonstrated the effects of weighted blankets on alleviating sleep disorders in patients with Alzheimer’s and easing the caregiver’s burden (45). In addition, the longer use time of weighted blankets in patients with dementia provided indirect evidence that weighted blankets may be beneficial (34).

#### 3.4.3 In patients with psychiatric disorders

Three studies were conducted on patients with different psychiatric disorders. A randomized controlled trial in Sweden showed that weighted blankets could improve insomnia, daytime symptoms, and activity levels in various psychiatric disorders (16). Another clinical trial concluded that weighted blankets could help patients in psychiatric hospitals better manage anxiety (38). A registry study also observed a statistically significant association between weighted blankets intervention and decreased use of common sleep medication in patients with psychiatric disorders (32).

#### 3.4.4 Others

Several studies focused on the impact of weighted blankets on other special populations. One exploratory study showed that parents of children with CHARGE syndrome found weighted blankets slightly effective in improving children’s sleep problems (24). The positive effects of weighted blankets were also found in children under general anesthesia, indicating that weighted blankets can be safely used for hospitalized children (46). Another crossover randomized controlled trial in 16 infants with neonatal withdrawal syndrome (NAS) found no adverse events during 67 30-minute sessions with weighted blanket, confirming the safety, feasibility, and efficacy of weighted blanket in reducing NAS symptoms (47). Only one study proved that using weighted blankets for 30 minutes could reduce anxiety better than standard care among cancer patients (37).

### 3.5 Comparison of weighted blankets and drug therapy

Although sleep drugs have been widely used in clinical practice, the side effects of drugs (e.g., drug resistance and drug tolerance) could rebound after drug withdrawal, making it not a good option. Also, the adverse outcomes caused by drug abuse were still great troubles for patients with sleep disorders (48, 49). For these reasons, sleep drugs are not recommended for treating chronic sleep disorders and anxiety disorders, or people over 65 years old (50). Weighted blankets were considered one of the non-pharmacological approaches to alleviate sleep disorders in patients with ASD without adverse effects (51). Several studies directly compared weighted blankets with drug therapy from the rate of prescription (32), perceived efficacy (24), and economic cost (34), respectively. First, a population-based register study of 1785 adult individuals with a psychiatric diagnosis directly compared the subscription of common sleep drugs (benzodiazepine receptor agonists, antihistamines, melatonin, and mirtazapine) before and after receiving a weighted blanket prescription, and found a significant association between weighted blanket use and the reduced use of common sleep drugs except melatonin that increased slightly (32). Second, however, in a cross-sectional study of children with CHARGE syndrome, their parents rated weighted blankets less effective than melatonin in improving their sleep quality. One possible reason is that melatonin is a common treatment that is more trusted by parents when recommended by doctors (24). Besides, the cost of weighted blankets as a prescription is higher than sleep drugs, which may lead to easier use and acceptance of sleep drugs. The average cost of a weighted blanket prescription for six months is just under €190, compared with sleep medications for the same period that was slightly below €86 in western Sweden. The higher cost of weighted blankets was mainly due to the more lengthy and complex prescription process and less to the material (34). In general, a weighted blanket is a non-drug supplement nearly without side effects, which might be safer than drug therapy in sleep intervention. Due to lower adverse effects but higher economic cost, the prescription pattern of weighted blankets should be revised and systemized to reduce the costs and better identify the target populations who may benefit from weighted blankets.

## 4 Discussion

Our review summarized all available studies examining the use and efficacy of weighted blankets in improving sleep quality, ADHD/ASD-related symptoms, negative emotions, and other related disorders in different populations, indicating a variability in the results, but a majority of studies showed positive results. Although the efficacy in individuals with sleep disorders and ADHD/ASD showed stronger supporting evidence, further validations are still needed due to the non-significant or negative results reported by a few studies. Meanwhile, due to the differences in the populations, outcomes, and intervention strategies (e.g., duration and frequency) in the included studies, particular caution is required when generalizing the findings to other populations.

### 4.1 Mechanisms of weighted blankets

**Figure 1:**
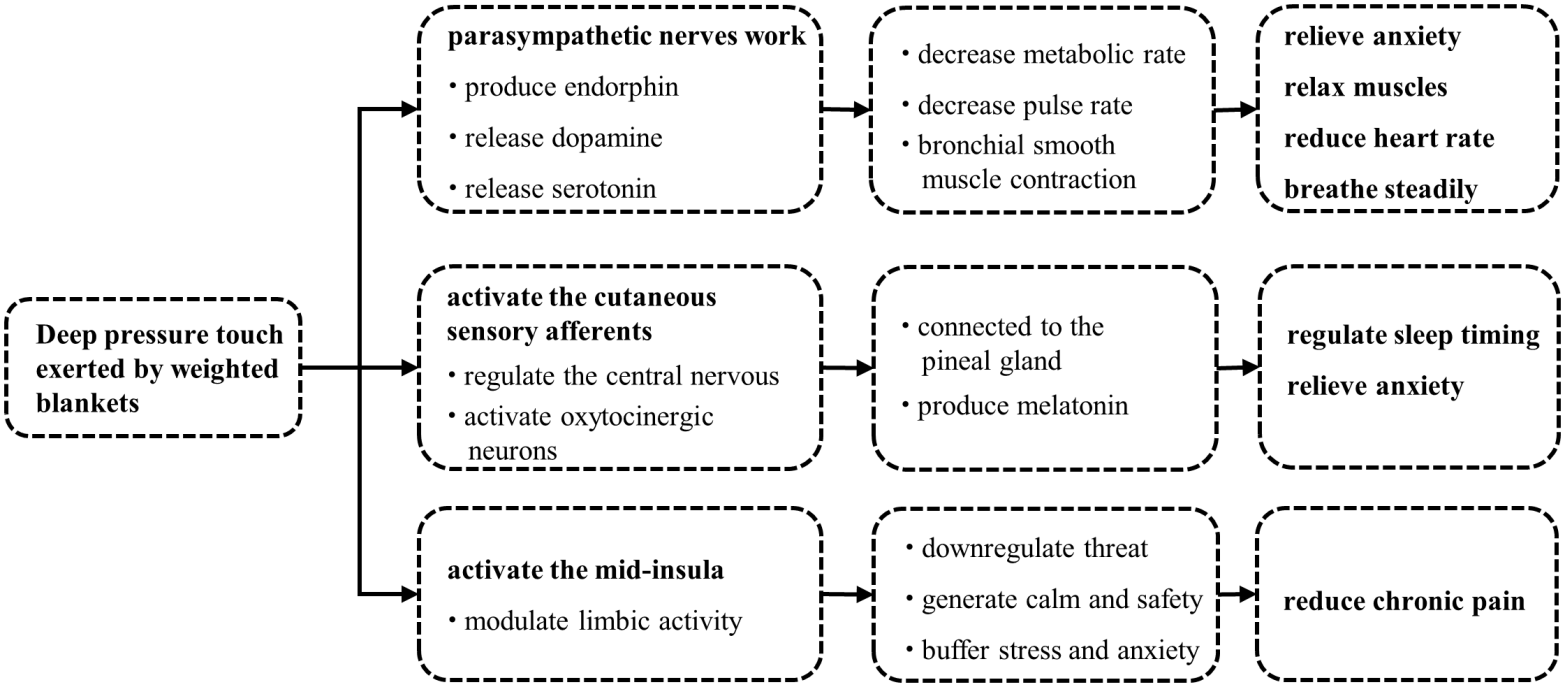
Mechanisms of weighted blankets.

Deep pressure touch (DPT) is the most recognized mechanism of weighted blankets by researchers (**Figure 1**) (52, 53). DPT is a form of tactile input which can be provided by holding, touching, embracing, stepping, and squeezing (14). A weighted blanket can cause changes in sensory nerve endings through continuous mechanical stimulations such as touch and pressure to the skin, leading to the opening of mechanically gated sodium channels, Na+ influx, and the generation of receptor potentials. The sympathetic nerve deals with the body’s decision to fight or flight; if it takes over, individuals can experience nervousness, anxiety, fear, irritability, poor sleep, and even digestive problems (54). The parasympathetic nervous system has a calming effect, which determines the “rest and digestion” of the body (55). A weighted blanket is a typical application of DPT, and can stimulate the parasympathetic nervous system, which has been supported by previous physiological studies (53, 56). When the parasympathetic nerve works, it can produce endorphins and release dopamine and serotonin (57), while endorphins and dopamine can result in reduced heart rate, relieved anxiety, relaxed muscles, and steady breath (58), and consequently, conducing to staying asleep. In a study of various tactile stimulations ending apnea in preterm infants, deep pressure stimulation was effective in helping preterm infants return to spontaneous breathing quickly (59).

Furthermore, a recent in-laboratory crossover study found that weighted blankets increased pre-sleep salivary concentrations of melatonin in young, healthy adults (25), providing new evidence for the sleep-promoting mechanisms of weighted blankets. Melatonin is released by the pineal gland and plays a critical role in sleep timing and anti-anxiety (60, 61). One explanation could be that the deep pressure provided by the weighted blankets activates cutaneous sensory afferents that transmit sensory information to the nucleus tractus solitarius through the spinal cord. The nucleus tractus solitarius projects to the paraventricular nucleus of the hypothalamus, which hosts parvocellular oxytocinergic neurons (62). They subsequently connect to the pineal gland to affect the release of melatonin (63). Notably, a previous study showed that a total loss of afferent sensory signals was related to a complete absence of nocturnal melatonin increase, reinforcing that the peripheral sensory nervous system plays a critical role in regulating the release of melatonin from central nervous system (64).

Interestingly, weighted blankets also contributed to relieving perceived bodily pain (41). This may result from several mechanisms. First, given that chronic pain is determined in part by social and/or emotional factors (65, 66), we speculate that the deep pressure provided by a weighted blanket may downregulate threat and generate calm and safety through activation of the insula, thereby alleviating stress and anxiety and ultimately reducing perception of bodily pain (67, 68). Second, the deep pressure can increase local tissue oxygenation and blood flow. These peripheral effects may account for the antinociceptive function of the weighted blankets (69, 70).Third, studies have reported that deep pressure sensation is transmitted by A-beta afferents (71), which was found to suppress laser pain in the same dermatome at the spinal cord level (72), suggesting that deep pressure provided by a weighted blanket might reduce bodily pain through A-beta stimulation.

### 4.2 Current research gaps and potential developments

This review reflects several current research gaps in weighted blankets therapy. First, the sample size of most existing studies did not reach 100, calling for more large-scale studies. Second, there was a lack of prospective, parallel and controlled studies, with many existing studies being poor clinical trials or observational studies, indicating a huge evidence gap. High-quality randomized controlled trials with longer intervention duration are required to strengthen the level of evidence. Third, sleep measurements were mostly subjective, hence, the results lack credibility. Objective measurements such as actigraphy covering multiple sleep outcomes should be applied. In addition, it is necessary to distinguish between subjective and objective effects of the weighted blankets. For instance, a review on non-traditional sleep treatments in children and young people reported that the subjects and caregivers using weighted blankets had positive feedbacks, despite a lack of significant differences in their objective sleep indicators (23). Forth, a few studies were relatively weak in statistical analysis, such as uncontrolled confounding factors, inappropriate use of statistical methods, etc. Moreover, several studies were qualitative, and these conclusions need to be further confirmed by empirical research with rigorous statistical methods. Fifth, the limited evidence of how weighted blankets work indicates a need for more research into the potential mechanisms. Future research could explore how weighted blankets can improve sleep through its effects on the brain using objective measurements such as electroencephalogram. Sixth, future studies could try to test the effects of weighted blankets in healthy people without insomnia or to test the non-inferiority of weighted blankets using a well-recognized sleep medication as a positive control.

### 4.3 Clinical implications

The efficacy of weighted blankets in sleep quality in different populations provides scientific recommendations for a non-pharmacotherapy of sleep disorders such as insomnia in clinical settings. Nevertheless, given the limited evidence available, the clinical application should be especially cautious. Clinicians should help patients make a reasonable decision by informing them of the uncertainty of the efficacy and other alternative approaches, considering the patients’ core needs and preferences. In practice, clinicians should evaluate whether the patients have characteristics unsuitable for using weighted blankets, such as a history of serious diseases including coronary heart disease, cerebral infarction, respiratory problems, and cancer; active abuse of sleeping pills; severe cognitive dysfunction, etc. Once a patient starts using, it is better for clinicians to monitor closely, collect the feedbacks in time, and discontinue immediately if the patient feels uncomfortable. It is important to note that weighted blankets can be dangerous, especially for children and the elderly. The higher weight of weighted blankets may cause pain, anxiety, and panic to a few children (33, 73). In general, children under 3 years of age or weighing less than 50 pounds should not use weighted blankets due to the risk of suffocation or entrapment. It is best for children to use a weighted blanket under the supervision of their parents and will need to go through a period of adaptation. We advise parents to avoid covering the children’s face with a blanket. For the elderly, it is worth emphasizing that they should have the physical capacity to remove the weighted blanket from the head if needed to avoid the risk of suffocation. If the blanket is too heavy that they have to struggle to get it off, they could end up injuring themselves. Therefore, if the elderly have frailty, limited mobility, or severe dementia, they should use the lightest weighted blanket possible after consulting medical doctors (45).

In summary, weighted blankets might be a safe and effective intervention for insomnia and various sleep disturbances and psychiatric disorders, with fewer side effects than drug therapy. More research is needed further to explore the application of weighted blankets in a wider range of populations. At present, deep pressure touch has been recognized as an essential mechanism of weighted blankets. Further studies aiming at identifying more mechanisms of weighted blankets are needed. The long-term safety and efficacy of weighted blankets must be further validated in high-quality, large-scale randomized controlled trials.

## Author contributions

JY: Methodology, Visualization, Writing – original draft, Writing – review & editing. ZY: Methodology, Writing – original draft. SS: Methodology, Visualization, Writing – original draft. KS: Investigation, Writing – review & editing. WC: Investigation, Writing – review & editing. LMZ: Investigation, Writing – review & editing. JX: Investigation, Writing – review & editing. QX: Investigation, Writing – review & editing. ZL: Conceptualization, Funding acquisition, Supervision, Writing – review & editing. JK: Investigation, Writing – review & editing. LSZ: Supervision, Writing – review & editing. YZ: Supervision, Writing – review & editing.
