# Supplementary figures and images for: The effect of weighted blankets on sleep and related disorders: a brief review
Source: Front Psychiatry. 2024 Apr 15;15:1333015. doi: 10.3389/fpsyt.2024.1333015 (PMC11056563; doi:10.3389/fpsyt.2024.1333015)

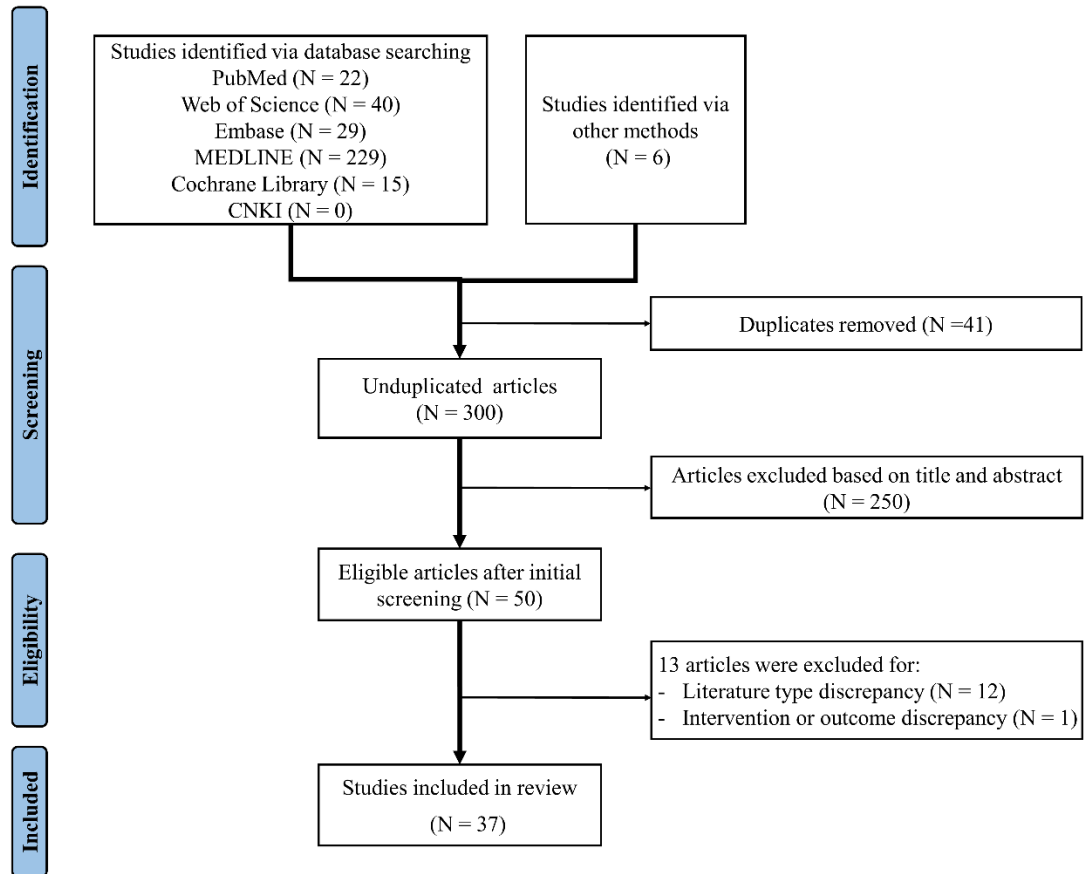

**eFigure 1. Study flow chart**

Supplement: Supplementary file 1 [file Image_1.pdf]
